# 3-(4-Bromo­phen­yl)-3-(4-hydr­oxy-6-oxo-1,6-dihydro­pyrimidin-5-yl)-*N*-[(*S*)-1-phenyl­ethyl]propanamide

**DOI:** 10.1107/S1600536809003821

**Published:** 2009-02-06

**Authors:** Ju-Hua Peng, Wen-Juan Hao, Shu-Jiang Tu

**Affiliations:** a Lianyungang Teachers’ College, Lianyungang 222006, People’s Republic of China; b School of Chemistry and Chemical Engineering, Xuzhou Normal University, Xuzhou 221116, People’s Republic of China

## Abstract

In the mol­ecule of the title compound, C_21_H_20_BrN_3_O_3_, the pyrimidine ring is oriented at dihedral angles of 80.87 (3) and 15.99 (3)°, respectively, to the pyrimidine and bromo­phenyl rings. The dihedral angle between the two benzene rings is 88.37 (3)°. In the crystal structure, inter­molecular N—H⋯O and O—H⋯N hydrogen bonds link the mol­ecules. A π–π contact between pyrimidine and phenyl rings [centroid–centroid distance = 3.776 (3) Å] may further stabilize the structure. The methine H and the methyl C and H atoms are disordered over two positions and were refined with occupancies of 0.522 (13) and 0.478 (13).

## Related literature

For general background, see: Johar *et al.* (2005 ▶); Janeba *et al.* (2005 ▶); Soloducho *et al.* (2003 ▶); Mathews & Asokan (2007 ▶); Lagoja (2005 ▶); Michael (2005 ▶); Erian (1993 ▶). For bond-length data, see: Allen *et al.* (1987 ▶). 
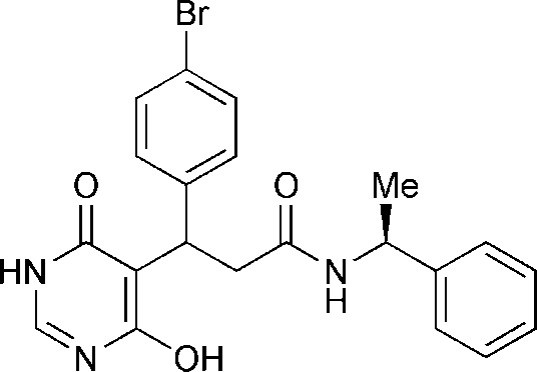


## Experimental

### 

#### Crystal data

C_21_H_20_BrN_3_O_3_
*M* *_r_* = 442.31
Triclinic, 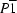

*a* = 7.147 (1) Å
*b* = 12.5010 (14) Å
*c* = 13.0940 (16) Å
α = 118.506 (2)°
β = 99.047 (1)°
γ = 93.074 (1)°
*V* = 1004.0 (2) Å^3^
*Z* = 2
Mo *K*α radiation
μ = 2.07 mm^−1^
*T* = 298 (2) K
0.20 × 0.18 × 0.17 mm

#### Data collection

Bruker SMART CCD area-detector diffractometer
Absorption correction: multi-scan (*SADABS*; Bruker, 1998 ▶) *T*_min_ = 0.682, *T*_max_ = 0.719
5285 measured reflections
3498 independent reflections
1810 reflections with *I* > 2σ(*I*)
*R*_int_ = 0.029

#### Refinement

*R*[*F* ^2^ > 2σ(*F* ^2^)] = 0.062
*wR*(*F* ^2^) = 0.162
*S* = 1.06
3498 reflections
265 parameters
H-atom parameters constrained
Δρ_max_ = 0.38 e Å^−3^
Δρ_min_ = −0.43 e Å^−3^

### 

Data collection: *SMART* (Bruker, 1998 ▶); cell refinement: *SAINT* (Bruker, 1998 ▶); data reduction: *SAINT*; program(s) used to solve structure: *SHELXS97* (Sheldrick, 2008 ▶); program(s) used to refine structure: *SHELXL97* (Sheldrick, 2008 ▶); molecular graphics: *SHELXTL* (Sheldrick, 2008 ▶); software used to prepare material for publication: *SHELXTL*.

## Supplementary Material

Crystal structure: contains datablocks global, I. DOI: 10.1107/S1600536809003821/hk2614sup1.cif

Structure factors: contains datablocks I. DOI: 10.1107/S1600536809003821/hk2614Isup2.hkl

Additional supplementary materials:  crystallographic information; 3D view; checkCIF report

## Figures and Tables

**Table 1:** Hydrogen-bond geometry (Å, °)

*D*—H⋯*A*	*D*—H	H⋯*A*	*D*⋯*A*	*D*—H⋯*A*
N1—H1⋯O3^i^	0.86	1.88	2.694 (3)	158
O2—H2⋯N2^ii^	0.82	1.87	2.681 (3)	170
N3—H3⋯O1^iii^	0.86	2.09	2.892 (3)	155

Symmetry codes: (i) 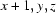; (ii) 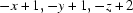; (iii) 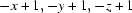.

**Fig. 1.**
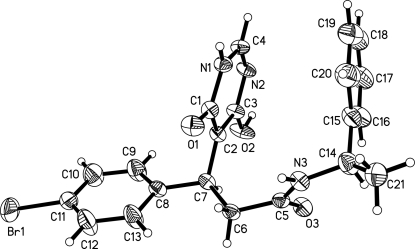
The molecular structure of the title molecule, with the atom-numbering scheme. Displacement ellipsoids are drawn at the 50% probability level.

## supplementary crystallographic information

### Comment

The pyrimidines and their derivatives as a class of extremely important heterocyclic compounds are used in a wide array of synthetic and industrial applications. Not only they are an integral part of the genetic materials, *viz*. DNA and RNA as nucleotides and nucleosides but also play critical roles especially in pharmaceutical fields (Johar *et al.*, 2005; Janeba *et al.*, 2005). Some pyrimidine derivatives can give stable and good quality nanomaterials having many important electrical and optical properties (Soloducho *et al.*, 2003; Mathews & Asokan, 2007), and also used as functional materials (Lagoja, 2005; Michael, 2005; Erian, 1993). We report herein the crystal structure of the title compound.

In the molecule of the title compound (Fig 1), the bond lengths (Allen *et al.*,  1987) and angles are within normal ranges. Rings A (N1/N2/C1-C4), B (C8-C13) and C (C15-C20) are, of course, planar, and they are oriented at dihedral angles of A/B = 80.87 (3)°, A/C = 15.99 (3)° and B/C = 88.37 (3)°.

In the crystal structure, intermolecular N—H···O and O—H···N hydrogen bonds (Table 1) link the molecules, in which they may be effective in the stabilization of the structure. The π-π contact between the pyrimidine and the phenyl rings, Cg1—Cg2 [where Cg1 and Cg2 are centroids of the rings A (N1/N2/C1-C4) and C (C15-C20), respectively] may further stabilize the structure, with centroid-centroid distance of 3.776 (3) Å.

### Experimental

The title compound was prepared by the reaction of 4-bromophenylidene-Meldrum's acid (1 mmol) with 6-hydroxypyrimidin-4(3*H*)-one (1 mmol) and (*S*)-1-phenylethanamine (1 mmol) at 373 K in glacial acetic acid under microwave irradiation (maximum power 250 W, initial power 100 W) for 18 min (yield; 85%, m.p. 551–553 K). Crystals suitable for X-ray analysis were obtained from an ethanol solution by slow evaporation.

### Refinement

Atoms C21, H21A, H21B, H21C and H14 were disordered over two positions. During the refinement process the disordered atoms were refined with occupancies of 0.522 (13) and 0.478 (13). H atoms were positioned geometrically, with O-H = 0.82 Å (for OH), N-H = 0.86 Å (for NH) and C-H = 0.93, 0.98, 0.97 and 0.96 Å for aromatic, methine, methylene and methyl H, respectively, and constrained to ride on their parent atoms, with U_iso_(H) = xU_eq_(C,N,O), where x = 1.5 for methyl and OH H and x = 1.2 for all other H atoms.

### Crystal data

**Table:**

C_21_H_20_BrN_3_O_3_	*Z* = 2
*M**_r_* = 442.31	*F*(000) = 452
Triclinic, *P*1	*D*_x_ = 1.463 Mg m^−^^3^
Hall symbol: -P 1	Melting point = 551–553 K
*a* = 7.147 (1) Å	Mo *K*α radiation, λ = 0.71073 Å
*b* = 12.5010 (14) Å	Cell parameters from 1260 reflections
*c* = 13.0940 (16) Å	θ = 3.1–25.2°
α = 118.506 (2)°	µ = 2.07 mm^−^^1^
β = 99.047 (1)°	*T* = 298 K
γ = 93.074 (1)°	Block, colorless
*V* = 1004.0 (2) Å^3^	0.20 × 0.18 × 0.17 mm

### Data collection

**Table:**

Bruker SMART CCD area-detector diffractometer	3498 independent reflections
Radiation source: fine-focus sealed tube	1810 reflections with *I* > 2σ(*I*)
graphite	*R*_int_ = 0.029
φ and ω scans	θ_max_ = 25.0°, θ_min_ = 1.8°
Absorption correction: multi-scan (*SADABS*; Bruker, 1998)	*h* = −8→8
*T*_min_ = 0.682, *T*_max_ = 0.719	*k* = −14→14
5285 measured reflections	*l* = −15→12

### Refinement

**Table:**

Refinement on *F*^2^	Primary atom site location: structure-invariant direct methods
Least-squares matrix: full	Secondary atom site location: difference Fourier map
*R*[*F*^2^ > 2σ(*F*^2^)] = 0.062	Hydrogen site location: inferred from neighbouring sites
*wR*(*F*^2^) = 0.162	H-atom parameters constrained
*S* = 1.06	*w* = 1/[σ^2^(*F*_o_^2^) + (0.0673*P*)^2^] where *P* = (*F*_o_^2^ + 2*F*_c_^2^)/3
3498 reflections	(Δ/σ)_max_ = 0.001
265 parameters	Δρ_max_ = 0.38 e Å^−^^3^
0 restraints	Δρ_min_ = −0.43 e Å^−^^3^

### Special details

**Table:**

Geometry. All e.s.d.'s (except the e.s.d. in the dihedral angle between two l.s. planes) are estimated using the full covariance matrix. The cell e.s.d.'s are taken into account individually in the estimation of e.s.d.'s in distances, angles and torsion angles; correlations between e.s.d.'s in cell parameters are only used when they are defined by crystal symmetry. An approximate (isotropic) treatment of cell e.s.d.'s is used for estimating e.s.d.'s involving l.s. planes.
Refinement. Refinement of *F*^2^ against ALL reflections. The weighted *R*-factor *wR* and goodness of fit *S* are based on *F*^2^, conventional *R*-factors *R* are based on *F*, with *F* set to zero for negative *F*^2^. The threshold expression of *F*^2^ > σ(*F*^2^) is used only for calculating *R*-factors(gt) *etc*. and is not relevant to the choice of reflections for refinement. *R*-factors based on *F*^2^ are statistically about twice as large as those based on *F*, and *R*- factors based on ALL data will be even larger.

### Fractional atomic coordinates and isotropic or equivalent isotropic displacement parameters (Å^2^)

**Table:**

	*x*	*y*	*z*	*U*_iso_*/*U*_eq_	Occ. (<1)
Br1	0.19659 (12)	−0.19766 (6)	0.27241 (8)	0.1005 (4)	
O1	0.5807 (5)	0.4123 (3)	0.5600 (3)	0.0571 (10)	
O2	0.3046 (5)	0.4229 (4)	0.8648 (3)	0.0636 (11)	
H2	0.3406	0.4455	0.9356	0.095*	
O3	0.0791 (5)	0.5971 (3)	0.7386 (3)	0.0556 (10)	
N1	0.7505 (6)	0.4888 (4)	0.7474 (4)	0.0428 (11)	
H1	0.8512	0.5063	0.7267	0.051*	
N2	0.6202 (6)	0.4935 (4)	0.9004 (4)	0.0478 (11)	
N3	0.3055 (6)	0.6437 (4)	0.6606 (4)	0.0475 (11)	
H3	0.3644	0.6160	0.6026	0.057*	
C1	0.5815 (7)	0.4342 (4)	0.6618 (5)	0.0408 (12)	
C2	0.4203 (7)	0.4109 (4)	0.7041 (4)	0.0394 (12)	
C3	0.4498 (7)	0.4424 (5)	0.8225 (4)	0.0417 (13)	
C4	0.7634 (7)	0.5149 (5)	0.8586 (5)	0.0450 (13)	
H4	0.8816	0.5508	0.9104	0.054*	
C5	0.1767 (7)	0.5650 (5)	0.6613 (4)	0.0420 (13)	
C6	0.1587 (8)	0.4333 (4)	0.5658 (4)	0.0445 (13)	
H6A	0.2353	0.4275	0.5088	0.053*	
H6B	0.0262	0.4026	0.5241	0.053*	
C7	0.2281 (7)	0.3561 (4)	0.6225 (4)	0.0429 (13)	
H7	0.1378	0.3585	0.6725	0.051*	
C8	0.2223 (7)	0.2209 (5)	0.5337 (5)	0.0457 (13)	
C9	0.2544 (10)	0.1396 (5)	0.5734 (6)	0.0734 (19)	
H9	0.2786	0.1682	0.6548	0.088*	
C10	0.2523 (11)	0.0158 (6)	0.4969 (7)	0.084 (2)	
H10	0.2783	−0.0369	0.5271	0.101*	
C11	0.2120 (9)	−0.0288 (5)	0.3769 (6)	0.0642 (17)	
C12	0.1737 (11)	0.0496 (6)	0.3352 (6)	0.088 (2)	
H12	0.1444	0.0200	0.2537	0.105*	
C13	0.1777 (11)	0.1740 (5)	0.4129 (5)	0.079 (2)	
H13	0.1496	0.2263	0.3826	0.094*	
C14	0.3576 (10)	0.7744 (6)	0.7498 (6)	0.0682 (17)	
H14	0.2380	0.7946	0.7776	0.082*	0.522 (13)
H14'	0.4428	0.8049	0.7146	0.082*	0.478 (13)
C15	0.4908 (10)	0.7923 (5)	0.8600 (6)	0.0644 (17)	
C16	0.4207 (11)	0.7902 (7)	0.9509 (7)	0.085 (2)	
H16	0.2890	0.7782	0.9449	0.102*	
C17	0.5439 (14)	0.8058 (7)	1.0516 (7)	0.099 (2)	
H17	0.4949	0.8039	1.1125	0.119*	
C18	0.7363 (16)	0.8239 (7)	1.0612 (8)	0.105 (3)	
H18	0.8182	0.8344	1.1289	0.126*	
C19	0.8110 (12)	0.8270 (7)	0.9733 (10)	0.108 (3)	
H19	0.9430	0.8394	0.9804	0.129*	
C20	0.6863 (11)	0.8114 (7)	0.8724 (8)	0.090 (2)	
H20	0.7365	0.8138	0.8121	0.108*	
C21	0.3839 (18)	0.8563 (10)	0.6965 (11)	0.072 (5)	0.522 (13)
H21A	0.2689	0.8443	0.6410	0.108*	0.522 (13)
H21B	0.4888	0.8360	0.6562	0.108*	0.522 (13)
H21C	0.4108	0.9408	0.7585	0.108*	0.522 (13)
C21'	0.211 (2)	0.8496 (13)	0.7670 (15)	0.096 (7)	0.478 (13)
H21D	0.1534	0.8433	0.6926	0.144*	0.478 (13)
H21E	0.2647	0.9336	0.8230	0.144*	0.478 (13)
H21F	0.1152	0.8226	0.7970	0.144*	0.478 (13)

### Atomic displacement parameters (Å^2^)

**Table:**

	*U*^11^	*U*^22^	*U*^33^	*U*^12^	*U*^13^	*U*^23^
Br1	0.0949 (6)	0.0471 (4)	0.1247 (8)	0.0142 (4)	0.0106 (5)	0.0183 (4)
O1	0.064 (3)	0.080 (3)	0.037 (2)	0.007 (2)	0.0134 (19)	0.035 (2)
O2	0.045 (2)	0.106 (3)	0.037 (2)	−0.023 (2)	−0.0018 (18)	0.040 (2)
O3	0.046 (2)	0.062 (2)	0.052 (2)	−0.0011 (19)	0.018 (2)	0.020 (2)
N1	0.036 (3)	0.052 (3)	0.040 (3)	0.003 (2)	0.008 (2)	0.023 (2)
N2	0.045 (3)	0.060 (3)	0.032 (3)	−0.008 (2)	−0.001 (2)	0.021 (2)
N3	0.051 (3)	0.051 (3)	0.039 (3)	0.001 (2)	0.014 (2)	0.020 (2)
C1	0.045 (3)	0.045 (3)	0.038 (3)	0.006 (3)	0.014 (3)	0.023 (3)
C2	0.045 (3)	0.040 (3)	0.034 (3)	−0.001 (2)	0.004 (2)	0.020 (3)
C3	0.039 (3)	0.052 (3)	0.035 (3)	−0.004 (3)	0.004 (3)	0.024 (3)
C4	0.036 (3)	0.058 (3)	0.039 (4)	0.003 (3)	0.002 (3)	0.025 (3)
C5	0.039 (3)	0.049 (3)	0.038 (3)	0.006 (3)	0.006 (3)	0.023 (3)
C6	0.050 (3)	0.047 (3)	0.034 (3)	0.002 (3)	0.001 (2)	0.020 (3)
C7	0.045 (3)	0.043 (3)	0.036 (3)	−0.001 (3)	0.005 (3)	0.018 (3)
C8	0.047 (3)	0.047 (3)	0.041 (3)	−0.002 (3)	0.003 (3)	0.022 (3)
C9	0.109 (6)	0.056 (4)	0.054 (4)	0.007 (4)	0.000 (4)	0.031 (4)
C10	0.110 (6)	0.055 (4)	0.081 (5)	0.012 (4)	0.002 (4)	0.034 (4)
C11	0.062 (4)	0.048 (3)	0.068 (5)	0.004 (3)	0.007 (3)	0.020 (4)
C12	0.130 (7)	0.057 (4)	0.059 (5)	0.007 (4)	0.000 (4)	0.020 (4)
C13	0.131 (6)	0.048 (4)	0.044 (4)	0.008 (4)	−0.006 (4)	0.020 (3)
C14	0.079 (5)	0.057 (4)	0.063 (4)	−0.005 (4)	0.005 (4)	0.030 (4)
C15	0.067 (5)	0.049 (3)	0.060 (4)	−0.004 (3)	0.007 (4)	0.017 (3)
C16	0.076 (5)	0.099 (6)	0.063 (5)	−0.004 (4)	0.009 (4)	0.029 (4)
C17	0.105 (7)	0.100 (6)	0.066 (6)	−0.007 (5)	0.000 (5)	0.028 (5)
C18	0.107 (8)	0.081 (5)	0.082 (7)	0.006 (5)	−0.016 (6)	0.016 (5)
C19	0.070 (6)	0.098 (6)	0.110 (8)	0.006 (5)	−0.005 (6)	0.024 (6)
C20	0.070 (5)	0.092 (5)	0.091 (6)	0.003 (4)	0.018 (5)	0.032 (5)
C21	0.071 (9)	0.059 (7)	0.088 (10)	0.001 (6)	0.007 (7)	0.042 (7)
C21'	0.094 (13)	0.066 (9)	0.104 (13)	0.014 (9)	−0.007 (10)	0.031 (9)

### Geometric parameters (Å, °)

**Table:**

Br1—C11	1.874 (6)	C10—H10	0.9300
O1—C1	1.225 (5)	C11—C12	1.352 (8)
O2—C3	1.314 (5)	C12—C13	1.389 (8)
O2—H2	0.8200	C12—H12	0.9300
O3—C5	1.239 (5)	C13—H13	0.9300
N1—C4	1.318 (6)	C14—C21'	1.418 (15)
N1—C1	1.391 (6)	C14—C21	1.507 (12)
N1—H1	0.8600	C14—C15	1.508 (9)
N2—C4	1.305 (6)	C14—H14	0.9800
N2—C3	1.355 (6)	C14—H14'	0.9800
N3—C5	1.315 (6)	C15—C16	1.373 (9)
N3—C14	1.467 (7)	C15—C20	1.375 (9)
N3—H3	0.8600	C16—C17	1.387 (10)
C1—C2	1.428 (7)	C16—H16	0.9300
C2—C3	1.381 (6)	C17—C18	1.358 (11)
C2—C7	1.495 (7)	C17—H17	0.9300
C4—H4	0.9300	C18—C19	1.357 (11)
C5—C6	1.500 (7)	C18—H18	0.9300
C6—C7	1.534 (6)	C19—C20	1.394 (11)
C6—H6A	0.9700	C19—H19	0.9300
C6—H6B	0.9700	C20—H20	0.9300
C7—C8	1.520 (7)	C21—H14'	0.8878
C7—H7	0.9800	C21—H21A	0.9600
C8—C9	1.360 (7)	C21—H21B	0.9600
C8—C13	1.372 (8)	C21—H21C	0.9600
C9—C10	1.382 (8)	C21'—H21D	0.9600
C9—H9	0.9300	C21'—H21E	0.9600
C10—C11	1.366 (9)	C21'—H21F	0.9600
			
C3—O2—H2	109.5	C11—C12—C13	120.6 (6)
C4—N1—C1	123.0 (4)	C11—C12—H12	119.7
C4—N1—H1	118.5	C13—C12—H12	119.7
C1—N1—H1	118.5	C8—C13—C12	121.2 (6)
C4—N2—C3	116.3 (4)	C8—C13—H13	119.4
C5—N3—C14	125.9 (5)	C12—C13—H13	119.4
C5—N3—H3	117.1	C21'—C14—N3	117.5 (8)
C14—N3—H3	117.1	C21'—C14—C21	69.8 (8)
O1—C1—N1	119.3 (4)	N3—C14—C21	113.2 (7)
O1—C1—C2	125.8 (5)	C21'—C14—C15	116.8 (9)
N1—C1—C2	114.9 (5)	N3—C14—C15	111.5 (5)
C3—C2—C1	117.2 (5)	C21—C14—C15	122.5 (7)
C3—C2—C7	122.2 (4)	N3—C14—H14	102.1
C1—C2—C7	120.5 (4)	C21—C14—H14	102.1
O2—C3—N2	116.5 (4)	C15—C14—H14	102.1
O2—C3—C2	119.0 (5)	C21'—C14—H14'	103.5
N2—C3—C2	124.6 (4)	N3—C14—H14'	102.5
N2—C4—N1	124.0 (5)	C15—C14—H14'	102.1
N2—C4—H4	118.0	C16—C15—C20	118.0 (7)
N1—C4—H4	118.0	C16—C15—C14	121.0 (6)
O3—C5—N3	121.8 (5)	C20—C15—C14	120.9 (7)
O3—C5—C6	121.8 (5)	C15—C16—C17	120.8 (7)
N3—C5—C6	116.3 (4)	C15—C16—H16	119.6
C5—C6—C7	109.1 (4)	C17—C16—H16	119.6
C5—C6—H6A	109.9	C18—C17—C16	119.9 (8)
C7—C6—H6A	109.9	C18—C17—H17	120.0
C5—C6—H6B	109.9	C16—C17—H17	120.0
C7—C6—H6B	109.9	C19—C18—C17	121.0 (8)
H6A—C6—H6B	108.3	C19—C18—H18	119.5
C2—C7—C8	111.5 (4)	C17—C18—H18	119.5
C2—C7—C6	112.3 (4)	C18—C19—C20	118.8 (8)
C8—C7—C6	114.0 (4)	C18—C19—H19	120.6
C2—C7—H7	106.1	C20—C19—H19	120.6
C8—C7—H7	106.1	C15—C20—C19	121.5 (8)
C6—C7—H7	106.1	C15—C20—H20	119.2
C9—C8—C13	117.0 (5)	C19—C20—H20	119.2
C9—C8—C7	119.6 (5)	C14—C21—H21A	109.5
C13—C8—C7	123.3 (5)	C14—C21—H21B	109.5
C8—C9—C10	122.2 (6)	C14—C21—H21C	109.5
C8—C9—H9	118.9	H14'—C21—H21C	114.6
C10—C9—H9	118.9	C14—C21'—H21D	109.5
C11—C10—C9	119.9 (6)	C14—C21'—H21E	109.5
C11—C10—H10	120.1	H21D—C21'—H21E	109.5
C9—C10—H10	120.1	C14—C21'—H21F	109.5
C12—C11—C10	118.9 (6)	H21D—C21'—H21F	109.5
C12—C11—Br1	120.8 (5)	H21E—C21'—H21F	109.5
C10—C11—Br1	120.2 (5)		
			
C4—N1—C1—O1	−179.8 (5)	C13—C8—C9—C10	3.2 (10)
C4—N1—C1—C2	1.4 (7)	C7—C8—C9—C10	−179.5 (6)
O1—C1—C2—C3	−179.8 (5)	C8—C9—C10—C11	−1.8 (11)
N1—C1—C2—C3	−1.0 (6)	C9—C10—C11—C12	−0.4 (11)
O1—C1—C2—C7	−0.6 (8)	C9—C10—C11—Br1	−176.9 (5)
N1—C1—C2—C7	178.1 (4)	C10—C11—C12—C13	0.9 (11)
C4—N2—C3—O2	−178.8 (5)	Br1—C11—C12—C13	177.4 (6)
C4—N2—C3—C2	1.0 (8)	C9—C8—C13—C12	−2.6 (10)
C1—C2—C3—O2	179.6 (4)	C7—C8—C13—C12	−179.8 (6)
C7—C2—C3—O2	0.5 (7)	C11—C12—C13—C8	0.7 (12)
C1—C2—C3—N2	−0.1 (8)	C5—N3—C14—C21'	−59.8 (11)
C7—C2—C3—N2	−179.3 (5)	C5—N3—C14—C21	−138.3 (7)
C3—N2—C4—N1	−0.7 (8)	C5—N3—C14—C15	78.9 (7)
C1—N1—C4—N2	−0.5 (8)	C21'—C14—C15—C16	51.4 (11)
C14—N3—C5—O3	2.8 (8)	N3—C14—C15—C16	−87.7 (8)
C14—N3—C5—C6	−175.3 (5)	C21—C14—C15—C16	133.6 (8)
O3—C5—C6—C7	−65.8 (6)	C21'—C14—C15—C20	−128.6 (10)
N3—C5—C6—C7	112.3 (5)	N3—C14—C15—C20	92.4 (7)
C3—C2—C7—C8	−108.8 (5)	C21—C14—C15—C20	−46.4 (10)
C1—C2—C7—C8	72.1 (6)	C20—C15—C16—C17	−0.5 (10)
C3—C2—C7—C6	121.9 (5)	C14—C15—C16—C17	179.5 (6)
C1—C2—C7—C6	−57.2 (6)	C15—C16—C17—C18	0.3 (12)
C5—C6—C7—C2	−50.3 (6)	C16—C17—C18—C19	0.0 (13)
C5—C6—C7—C8	−178.3 (4)	C17—C18—C19—C20	0.1 (13)
C2—C7—C8—C9	60.7 (7)	C16—C15—C20—C19	0.6 (11)
C6—C7—C8—C9	−170.9 (5)	C14—C15—C20—C19	−179.5 (7)
C2—C7—C8—C13	−122.2 (6)	C18—C19—C20—C15	−0.4 (12)
C6—C7—C8—C13	6.2 (8)		

### Hydrogen-bond geometry (Å, °)

**Table:**

*D*—H···*A*	*D*—H	H···*A*	*D*···*A*	*D*—H···*A*
N1—H1···O3^i^	0.86	1.88	2.694 (3)	158
O2—H2···N2^ii^	0.82	1.87	2.681 (3)	170
N3—H3···O1^iii^	0.86	2.09	2.892 (3)	155

Symmetry codes: (i) *x*+1, *y*, *z*; (ii) −*x*+1, −*y*+1, −*z*+2; (iii) −*x*+1, −*y*+1, −*z*+1.

## References

[bb1] Allen, F. H., Kennard, O., Watson, D. G., Brammer, L., Orpen, A. G. & Taylor, R. (1987). *J. Chem. Soc. Perkin Trans. 2*, pp. 1–S19.

[bb2] Bruker (1998). *SMART*, *SAINT* and *SADABS* Bruker AXS, Inc., Madison, Wisconsin, USA.

[bb3] Erian, A. W. (1993). *Chem. Rev.* **93**, 1991–2005.

[bb4] Janeba, Z., Balzarini, J., Andrei, G., Robert Snoeck, R., De Clercq, E. & Robins, M. J. (2005). *J. Med. Chem.* **48**, 4690–4696. 10.1021/jm050291s 16000005

[bb5] Johar, M., Manning, T., Kunimoto, D. Y. & Kumar, R. (2005). *Bioorg. Med. Chem.* **13**, 6663–6671. 10.1016/j.bmc.2005.07.046 16140016

[bb6] Lagoja, I. M. (2005). *Chem. Biodivers.* **2**, 1–50. 10.1002/cbdv.200490173 17191918

[bb7] Mathews, A. & Asokan, C. V. (2007). *Tetrahedron*, **63**, 7845–7849.

[bb8] Michael, J. P. (2005). *Nat. Prod. Rep.* **22**, 627–646. 10.1039/b413750g 16193160

[bb9] Sheldrick, G. M. (2008). *Acta Cryst.* A**64**, 112–122. 10.1107/S0108767307043930 18156677

[bb10] Soloducho, J., Doskocz, J., Cabaj, J. & Roszak, S. (2003). *Tetrahedron*, **59**, 4761–4766.

